# CircRNA FAT1 Regulates Osteoblastic Differentiation of Periodontal Ligament Stem Cells via miR-4781-3p/SMAD5 Pathway

**DOI:** 10.1155/2021/5177488

**Published:** 2021-12-29

**Authors:** Yu Ye, Yue Ke, Liu Liu, Tong Xiao, Jinhua Yu

**Affiliations:** ^1^Jiangsu Key Laboratory of Oral Diseases, Nanjing Medical University & Department of Endodontic, Affiliated Hospital of Stomatology, Nanjing Medical University, Nanjing, China; ^2^Institute of Stomatology, Nanjing Medical University, Nanjing, China; ^3^Jiangsu Province Engineering Research Center of Stomatological Translational Medicine, Nanjing, China

## Abstract

The ability of human periodontal ligament stem cells (PDLSCs) to differentiate into osteoblasts is significant in periodontal regeneration tissue engineering. In this study, we explored the role and mechanism of circRNA FAT1 (circFAT1) in the osteogenic differentiation of human PDLSCs. The proliferation capacity of PDLSCs was evaluated by EdU and CCK-8 assay. The abilities of circFAT1 and miR-4781-3p in regulating PDLSC differentiation were analyzed by western blot, reverse transcription-polymerase chain reaction (RT-PCR), alkaline phosphatase (ALP), and Alizarin red staining (ARS). A nucleocytoplasmic separation experiment was utilized for circFAT1 localization. A dual-luciferase reporter assay confirmed the binding relationship between miR-4781-3p and circFAT1. It was showed that circFAT1 does not affect the proliferation of PDLSCs. The osteogenic differentiation of PDLSCs was benefited from circFAT1, which serves as a miRNA sponge for miR-4781-3p targeting SMAD5. Both knockdown of circFAT1 and overexpression of miR-4781-3p suppressed the osteogenic differentiation of PDLSCs. Thus, circFAT1 might be considered as a potential target of PDLSCs mediated periodontal bone regeneration.

## 1. Introduction

Periodontitis is a chronic oral infectious disease characterized by the disruption of periodontal supporting tissues integrity, including the destruction of alveolar bone, periodontal ligament (PDL), and cementum [1]. As one of the most common infection-driven diseases, Periodontitis can affect 90% of the global population [2]. Periodontitis can lead to loss of periodontal attachment and, if left untreated, can eventually lead to early tooth loss [3]. At present, the biggest challenge in the treatment of periodontitis is periodontal regeneration. Human periodontal ligament stem cells (PDLSCs) are mesenchymal stem cells (MSCs) derived from tooth tissues with high osteogenesis potential. Therefore, studying the molecular mechanism of osteogenic differentiation of PDLSCs is the keystone of the clinical application of tooth regeneration and osteogenic tissue engineering.

Noncoding RNA (ncRNA) is a kind of RNA transcribed from the genome, which can function at the RNA level rather than traditionally encode protein [4]. ncRNA can be classified into three types: (1) microRNA (miRNAs), siRNAs, and new noncoding small RNAs (piRNAs) with a length less than 50 NT; (2) the length ranges from 50 to 500 NT, including ribosomal RNA (rRNA) and transfer RNA (tRNA); and (3) longer than 500 NT, including long noncoding RNAs (lncRNAs) and circular RNAs (circRNAs) [4, 5]. miRNA is an endogenous noncoding small single-stranded RNA with a length of about 22 nucleotides [6]. It is worth noting that miRNAs participate in bone metabolism by acting on target genes related to osteogenic differentiation. Many miRNAs have been proved to participate in the osteogenic differentiation of PDLSCs [7–9].

The TGF-*β* signaling pathway is an essential pathway for the osteogenic differentiation procession. It relies on multiple SMAD proteins, such as receptor-regulated SMAD (R-SMAD), common SMAD (co-SMAD), and inhibitory SMAD (I-SMAD) [10]. SMAD family member 5 (SMAD5) is a R-SMAD protein. As a transcription factor, it takes part in the osteogenic differentiation of BMSCs [11]. When osteogenic signals are transmitted to the cytoplasm of BMSCs, phosphorylated-SMAD5 is directed to the nucleus and then regulates the expression of osteogenesis-related target genes [10]. The nuclear translocation of SMAD5 is key to osteogenesis signal transduction. LncTUG1 may inhibit the osteogenic differentiation of bone marrow mesenchymal stem cells (BMSCs) by targeting SMAD5 [12]. miR-24-3p targets SMAD5 to promote the osteogenic potential of PDLSCs [13]. miR-21, miR-17-5p, and miR-106b-5p inhibit bone formation by targeting SMAD5 [14, 15]. In another study, miR-222-3p depressed osteogenic differentiation of BMSCs, revealing the regulation of the SMAD5-RUNX2 signal axis [11].

CircRNAs are charactered by a special structure of continuous covalent closed loop, which has higher conservation and stability [16, 17]. CircRNAs have been applied in clinical treatment or disease diagnosis as biomarkers or targets [18]. Evidence has been demonstrated that circRNAs played a key role in developing numerous diseases by regulating key steps such as gene transcription, translation, and splicing [19, 20]. Studies on the accumulation of circRNAs revealed their important role in bone metabolism-related diseases [21]. Recently, circRNAs are discovered to be involved in maintaining the pluripotency of human embryonic stem cells (ESCs) [22], self-renewal ability of intestinal stem cells [23], the differentiation potential of osteoblasts and osteoclasts [24, 25], and even the rat liver regeneration [26]. So far, circRNAs have been well known as miRNA sponges. Thus, circRNAs work as posttranscriptional regulators, sponging with miRNAs and producing important biological effects [27]. Accumulating evidence shows that circRNAs play a nonnegligible role in many diseases, including periodontitis. Acting as a miR-7 sponge to upregulate Krueppel-like factor 4 (KLF4) expression, circCDR1 promoted PDLSCs stemness [28]. circCDK8 inhibited the osteogenic differentiation of PDLSCs by triggering autophagy activation in a hypoxic microenvironment [29].

CircRNA FAT1 (circFAT1), as a relatively new circRNA, has been reported to increase cell stemness of cancer cells through upregulation of miR-21 [30]. In breast cancer, circFAT1 may regulate miR-525-5P/SKA1 resistance through Notch and Wnt pathways, providing a potential target for breast cancer treatment [31]. circFAT1 has not been reported in any published literature about its effect on the osteogenic differentiation of PDLSCs. In our previous study, it was found that the expression of circFAT1 decreased in the hypoxic microenvironment, while the osteogenic differentiation ability of PDLSCs decreased. Thus, it can be inferred that circFAT1 may be positively correlated with the osteogenic differentiation of PDLSCs. miR-4781-3p was only found to be upregulated in Alzheimer's disease patients [32]. At present, there is no more literature report, and its exact role and mechanism in osteogenesis are still unclear. Sequencing results and preliminary experimental results showed that there is a binding relationship between circFAT1/miR-4781-3p/SMAD5, which may be involved in regulating the osteogenic differentiation of PDLSCs. Combined with bioinformatics predictions, this study investigates the mechanism by which circFAT1 may act as a sponge of miR-4781-3p to regulate SMAD5 and then affect the osteogenic differentiation of PDLSCs. It is expected to provide a new therapeutic target for exploring the periodontal regeneration mediated by PDLSCs.

## 2. Materials and Methods

### 2.1. Animals

Male SD rats (5 weeks old) were purchased from the experimental animal center of Nanjing Medical University and raised at the SPF level. All animal experiments were conducted according to the regulations of the ethics committee of Nanjing Medical University (IACUC-2010051). SD rats were anesthetized by intraperitoneal injection (1% sodium barbital) to establish a skull defect model. Make a 10 mm incision in the rat head and perform a trephine osteotomy on the cranial platform. Make two 5 mm diameter holes symmetrically on both sides of the midline of the skull. Implant the cell mass and the corresponding control group into the pores. Then, suture the wound. At week 8, skull tissue was collected for further analysis.

### 2.2. Microcomputed Tomography (micro-CT), Hematoxylin-Eosin (H&E), and Masson Staining

The rats were sacrificed eight weeks after surgery, and the cranial tissue was collected. The tissues were fixed with 4% paraformaldehyde (PFA) for one week and 75% ethanol for micro-CT evaluation. 3D images of the mineralized tissues were reconstructed using Sky scan software. The bone volume/tissue volume (BV/TV) of each sample was collected for analysis. After micro-CT evaluation, the tissues were demineralized in 14% EDTA solution for eight weeks, dehydrated by an automatic dehydrator, and embedded in paraffin. Paraffin sections were cut into tissue sections 5 mm thick for H&E and Masson staining.

### 2.3. Tissue Collection and Cell Culture

The premolars of healthy people extracted due to orthodontics were collected. Scrape the periodontal ligament tissue in the 1/3 area of the root, transfer it into a 10 cm sterile dish, and add *α*-minimum primary medium (*α*-MEM, GIBCO, California, USA) to keep the tissue moist, sharply separate about 1m^3^ tissue blocks of periodontal ligament tissue, lay the separated tissue blocks in culture flask at a spacing of 1 mm, inverted the flask, and add *α*-MEM (including 100 ml/L fetal bovine serum, 100u/ml penicillin, and 100 *μ*g/ml streptomycin). The culture flask was placed in an incubator at 37°C and 5% CO_2_ for culture. Turn over the tissue after 4 hours. The solution was changed every three days. The cells grew up to 80% and were sub cultured when confluence. A monoclonal screening method was adopted to purify PDLSCs. 3-5 generations of PDLSCs were cultivated for the follow-up experiments. The Ethics Committee of Nanjing Medical University School approved the relevant experiments (NJMU-2018202).

### 2.4. Adipogenic Differentiation

PDLSCs in the logarithmic growth stage were inoculated into the culture dish following the cell density of 2 × 10^4^ cells/cm^2^. The cells were cultured at 37°C, 5% CO_2_ environment to the confluence of 90-100%, the supernatant was discarded, and the adipogenic induction differentiation medium induction solution (Cyagen, Guangzhou, China) was added. After three days, the culture medium was replaced with an adipogenic differentiation medium induction solution. After one day of culture, it was replaced with adipogenic differentiation medium maintenance solution for three days. The cells were induced for 14-21 days according to the above liquid exchange frequency; then, the medium was aspirated. After washing once with 1 × PBS, PDLSCs were fixed with 4% PFA solution at room temperature for 30-60 minutes, and oil red O staining was performed.

### 2.5. Chondrogenic Differentiation

Transfer 3 × 10^5^ PDLSCs to a 15 ml centrifuge tube and centrifuge at 250 g for 4 minutes. Discard the supernatant, add 0.5 ml of chondrogenic differentiation medium basal solution, resuspend the cells, and centrifuge at 150 g for 5 minutes. Carefully discard the supernatant, add 0.5 ml chondrogenic differentiation medium induction solution (Cyagen, Guangzhou, China), resuspend the cells, and centrifuge at 150 g for 5 minutes. Unscrew the 15 ml centrifuge tube cap slightly and place it at 37°C, 5% CO_2_ incubator. After 24 hours, observe the deformation and accumulation of the cell pellets. The cell mass was transferred to a 24-well plate. Replace with induction solution every two days. After 21 days, the cartilage balls were fixed and sliced for Alcian blue staining.

### 2.6. Plasmid and siRNA Transfection

PDLSCs were inoculated in medium dishes and transfected when the fusion rate reached 50-60%. The transfected siRNA and plasmid were constructed by the company (RiboBio, Guangzhou, China). miR-4781-3p mimics (mimics, 50 nm), mimic negative control (NC, 50 nM), miR-4781-3p inhibitor (inhibitor, 100 nM), and inhibitor negative control (iNC, 100 nM) were mixed with Ribofect™ CP Kit (RiboBio, Guangzhou, China). Cells were stimulated for 24-72 hours. Similarly, PDLSCs transfected with circFAT1, SMAD5 siRNA, and negative controls (si-FAT1, si-SMAD5, NC, 100 nM) were performed similar operations. Luciferase reporter plasmids of circFAT1 and miR-4781-3p were constructed by predicting the binding sites. Lipofectamine 2000 was used as a transfection agent to transfect HEK-293 T cells.

### 2.7. Cell Proliferation Assay

Different stimuli were adopted to treat PDLSCs and then add 100 *μ*l of cell suspension in a 96-well plate. Incubate the culture plate for 0, 1, 3, 5, and 7 days. Add 10 *μ*l of CCK8 solution to each well and incubate the culture plate in the incubator for 1-4 hours. Measure the absorbance at 450 nm with a microplate reader. EdU was detected by Cell-Light™ EdU Apollo®567 *In Vitro* Imaging Kit (RiboBio, Guangzhou, China). The transfected PDLSCs were seeded in glass slides. After labeling with EdU, fixing the cells, staining with Apollo, and staining DNA with Hoechst 33342, the cells were observed with a fluorescence microscope. ImageJ software was used to calculate cell DNA replication efficiency.

### 2.8. Flow Cytometry

After the cells were treated differently, they were digested with trypsin without EDTA (Beyotime, Shanghai, China) and collected. The cells were resuspended in PBS and centrifuged twice, then resuspended in 100 *μ*L of PBS, and added with CD34, CD45, CD29, CD90, and CD105 surface molecule antibodies. After incubating on ice in the dark for 30 minutes, the cells were centrifuged with PBS. Then, the supernatant was removed, and flow cytometry analysis (BD Biosciences, CA, USA) was performed.

### 2.9. Quantitative Real-time RT-PCR (RT-qPCR)

TRIzol (Invitrogen, CA, USA) method was applied to extract RNA from cells. Then, the RNA was reverse transcripted to cDNA by a reverse transcription kit. Design the corresponding primers. Use the CHAMQ Universal SYBR qPCR Master Mix (Vazyme, Nanjing, China) reagent to set up the related program on the ABI QuantStudio 7 fluorescence quantitative PCR instrument (Applied Biological System) according to the instructions. The primer list is shown in Table 1.

### 2.10. Western Blot Analysis

The cell lysate was used to lyse the cells, the supernatant was taken after centrifugation, and the protein loading buffer was added; then, the protein sample was boiled. Perform electrophoresis experiments with 10% SDS-PAGE gel at 70 V constant pressure and transfer membrane at 300 mA constant current. After sealing with 5% milk for 2 hours, add primary antibody diluent (anti-COL1A (Proteintech, USA), anti-COL3A (Proteintech, USA), anti-RUNX2 (ABCAM, UK), anti-OSX (ABCAM, UK), anti-SMAD5 (Proteintech, USA), and anti-GAPDH (Cell signaling Technology, USA)). After 4°C overnight, protein bands were obtained by chemiluminescence gel imaging system. Then, the grey value analysis was performed.

### 2.11. Alkaline Phosphatase (ALP) Activity Assay and Alizarin Red Staining (ARS)

After seven days of induction of PDLSCs by adding mineralization induction solution, the ALP activity was detected, and ALP staining was performed. The alkaline phosphatase detection kit (Jiancheng, Nanjing, China) and the alkaline phosphatase color reagent kit (Beyotime, Shanghai, China) were used for detection. After 14 days of induction, 4% PFA was added to fix PDLSCs and then wash the cells with PBS 3 times. The cells were observed under the microscope after being incubated with Alizarin Red dye solution (40 mM, pH = 4.2, Sigma-Aldrich) for at least 1 hour. Cetylpyridine chloride (CPC, 100 mM) was used to dissolve calcified nodules, and the relative calcium mass was calculated according to the absorbance at 562 nm.

### 2.12. Immunofluorescence Staining

The treated PDLSCs were digested and placed on the cell slides. The next day, the culture medium was discarded and fixed with 4% PFA. Perforate the cells with Triton X-100 (Beyotime, Shanghai, China) for 12 minutes. Then, the cells were treated with block goat serum for 2 hours at 37°C, washed with PBS 3 times. Then, incubate the cells overnight at 4°C with ALP antibody diluted at 1 : 100, and the liquid with fluorescent secondary antibody was changed at room temperature in the dark for 2 hours. Phalloidin (Yeasen, Shanghai, China) was added to the cells for 30 minutes. DAPI (Beyotime, Shanghai, China) was dyed for 90 seconds for nuclear staining. Then, it was observed under a fluorescence microscope (Leica, Germany).

### 2.13. Nucleocytoplasmic Separation

Isolate cytoplasmic cytonuclear RNA from cells according to the manufacturer's instructions. Cell precipitation was collected after being digested and washed. Add 400 *μ*L Cell Separation Buffer to the cell precipitation. After being incubated for 10 minutes, the mixture was centrifugated at 500 × g for 1 minute to separate the cytoplasm and nucleus. Then, the nuclear residue was dissolved in a Cell Disruption Buffer. The nuclear and cytoplasmic samples were mixed thoroughly up with 2 × Lysis/Binding Solution at RT. Anhydrous ethanol was added to each sample, and the mixture was centrifuged for 1 minute. Wash solutions 1 and 2/3 were utilized to wash the samples. RNA from cytoplasm and nucleus were separately added to the preheated eluent and then collected for RT-PCR analysis.

### 2.14. RNA Fluorescence In Situ Hybridization (FISH)

PDLSCs were cultured on confocal plates. Then, after fixation with 4% PFA, the cells were washed with PBS and precooled with 0.5% Triton X-100 for 5 minutes at 4°C. Each well was prehybridized with 500 *μ*L prehybridization buffer at 37°C for 30 minutes. The FISH probe mixture or internal reference probe was then added to the 200 *μ*L preheated hybridization buffer. PDLSCs were then incubated overnight in darkness at 37°C in a hybridization buffer containing FISH probes. The cells were washed three times with the hybridized lotion at a concentration gradient of 42°C for 5 minutes, and DAPI has stained again in the dark for 10 minutes. Wash three times and use PBS to remove excess liquid. The cell photographs were taken with LSM 710 confocal microscope (Leica, Germany).

### 2.15. Dual-Luciferase Reporter Assay

miR-4781-3p and negative control were transfected into HEK-293 T cells. 100 ng circFAT1 or SMAD5 wild-type reporter plasmid and 20 ng renilla luciferase (RL) reporter plasmid was simultaneously transfected into HEK-293 T cells. Set circFAT1 or SMAD5 reported plasmid mutations as the control. After 48 hours, a dual-luciferase reporter gene detection kit (Promega, Madison, USA) was used to detect luciferase activity.

### 2.16. Statistical Analysis

Each experiment was repeated three times or more. Statistical analyzes were conducted with SPSS 17.0. *t*-test or one-way analysis of variance was used to the analyzed difference among groups. *P* < 0.05 was considered statistically different.

## 3. Results

### 3.1. Identification of PDLSCs and Verification of PDLSCs Multidirectional Differentiation Ability

**Figure 1:**
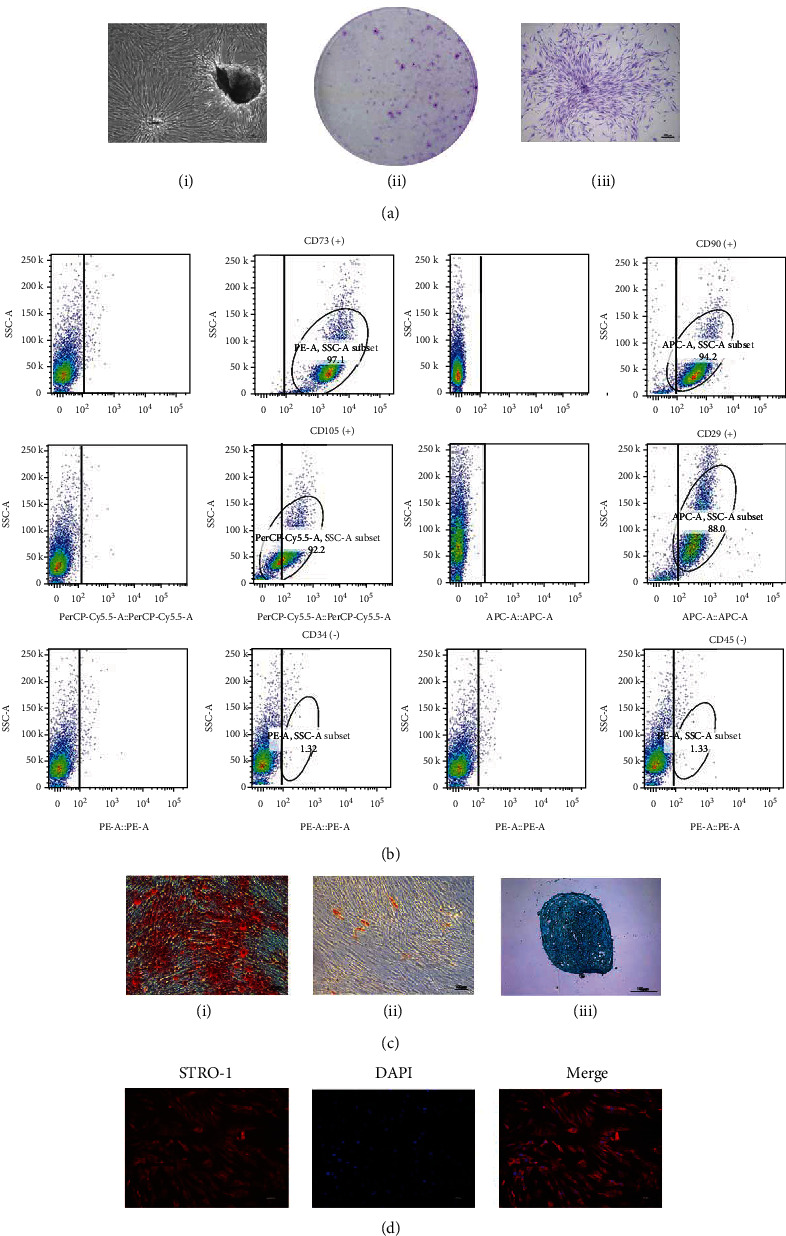
Identification of PDLSCs and verification of PDLSC multidirectional differentiation ability. (a): (I) Primary cells migrated from PDL tissues on day 3, and 80% confluence was observed on day 12 (scale bar: 100 *μ*m). (II, III) Cell colonies formed by PDLSCs observed in the dish and an amplified image of a representative colony captured by a microscope (scale bar: 100 *μ*m). (b) Flow cytometry results showed that CD29, CD73, CD90, and CD105 were positive in PDLSCs. In addition, both CD45 and CD34 were negative. (c) Multiple differentiation potentials of PDLSCs (from left to right: osteogenesis, adipogenesis, chondrogenesis; scale bar: 100 *μ*m). (d) Immunofluorescence assay revealed that cultured PDLSCs were positive for STRO-1 (scale bar: 100 *μ*m).

1. Primary PDLSCs crawled out from around the tissue, mostly long spindle-shaped and growing densely (Figure 1(a)). After screening the cells cultured by the monoclonal method, multiple scattered cell colonies similar to clones could be seen (Figures 1(a)–1(c)). Flow cytometry results showed that CD29, CD73, CD90, and CD105 were positive in PDLSCs. In addition, both CD45 and CD34 were negative (Figure 1(b)).
2. Characteristic staining was performed, respectively, to prove that PDLSCs had the ability of multidirectional differentiation into osteogenic, adipogenic, and chondrogenic (Figure 1(c)). Immunofluorescence results showed positive expression of STRO-1 (Figure 1(d)).

### 3.2. circFAT1 Does Not Affect PDLSCs Proliferation

**Figure 2:**
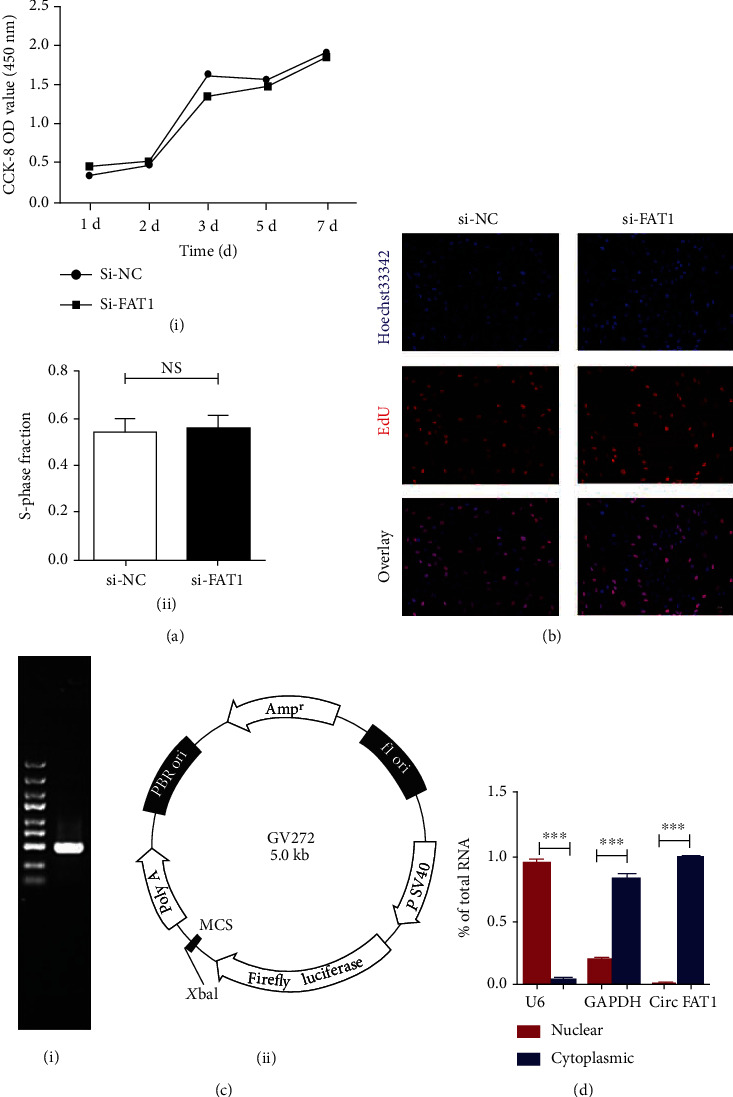
circFAT1 does not affect PDLSCs proliferation. (a) Cell proliferation capability influenced by circFAT1 was detected at 450 nm with CCK-8 assay. (I): CCK-8 results showed that circFAT1 had no significant effect on the proliferation of PDLSCs; (II): EdU assay further confirmed that circFAT1 had no significant effect on the proliferation of PDLSCs. (b) EdU-positive PDLSCs influenced by circFAT1 were detected by EdU kit (scale bar: 200 *μ*m). EdU (red), Hoechst333 (blue). (c) PCR gel electrophoresis verified that the primers were located at a single band of 449 kb. The cyclization site of circFAT1 was determined by plasmid vector construction. (d) RNA nucleocytoplasmic isolation experiments verified that circFAT1 was mainly located in the cytoplasm of cells. ^∗^*P* < 0.05, ^∗∗^*P* < 0.01, and ^∗∗∗^*P* < 0.001. EdU: 5-ethynyl-2-deoxyuridine.

1. CCK8 assay verified that PDLSCs transfection of circFAT1 siRNA had no significant effect on proliferation (Figure 2(a)). DNA replication ability of cells was detected by EdU assay, which further confirmed that the effect of circFAT1 on the proliferation of PDLSCs is not significant (Figures 2(a) and 2(b)).
2. PCR gel electrophoresis verified that the primers were located at a single band of 449 kb. The cyclization site of circFAT1 was determined by plasmid vector construction (Figure 2(c)) The results of RNA nucleocytoplasmic isolation proved that circFAT1 was mostly distributed in the cytoplasm (Figure 2(d)).

### 3.3. circFAT1 Silencing Inhibits Osteoblastic Differentiation Potential of PDLSCs

**Figure 3:**
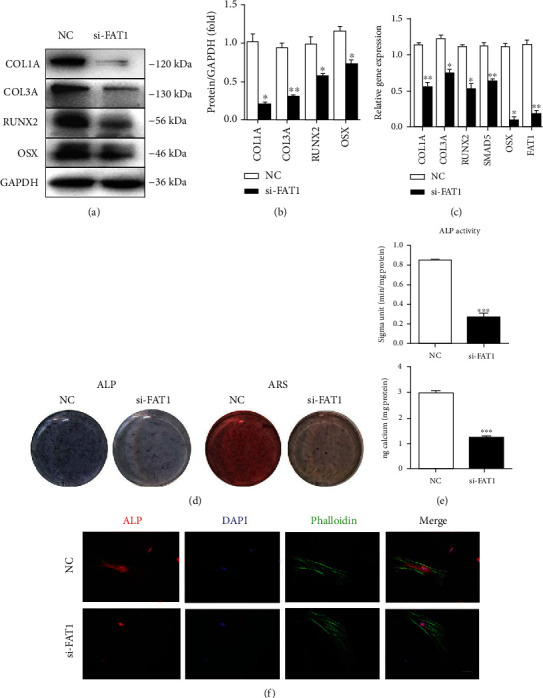
circFAT1 silencing inhibits osteoblastic differentiation potential of PDLSCs. (a)–(c) Western blot and RT-qPCR showed that osteogenic markers (RUNX2, OSX, COL1A, and COL3A) were downregulated in the circFAT1 siRNA-treated PDLSCs (si-FAT1 group). (d, e) Silence of circFAT1 reduced the ALP activity of PDLSCs 7 days after osteogenic induction. ARS showed the mineralization of PDLSCs was decreased significantly in the si-FAT1 group after osteogenic induction for 14 days. (f) Immunofluorescence experiments confirmed the decreased expression of ALP in the si-FAT1 group. ^∗^*P* < 0.05, ^∗∗^*P* < 0.01, and ^∗∗∗^*P* < 0.001.

To detect whether circFAT1 affects the osteogenic differentiation potential of PDLSCs, cells were transfected with NC or circFAT1 siRNA (si-FAT1), respectively. Western blot and RT-qPCR showed that biomarkers of osteogenesis (RUNX2, OSX, COL1A, and COL3A) were downregulated, and RT-qPCR results showed that the *circFAT1* expression was decreased (Figures 3(a)–3(c)).

The osteogenic induction medium was used for culturing two groups of cells, respectively. ARS confirmed that the mineralization nodules in the si-FAT1 group were decreased, and the calcium content calculated by the CPC assay was significantly decreased (*P* < 0.001) on 14 days. ALP activity was detected after seven days, which was used as an indicator for early observation of the osteogenic differentiation ability of PDLSCs. We found that the si-FAT1 group expresses less ALP, and meanwhile, the activity of ALP decreased significantly (*P* < 0.001) (Figures 3(d) and 3(e)). Immunofluorescence experiments confirmed the reduced expression of ALP in the si-FAT1 group (Figure 3(f)).

### 3.4. SMAD5 Regulates the Osteoblastic Differentiation Ability of PDLSCs

**Figure 4:**
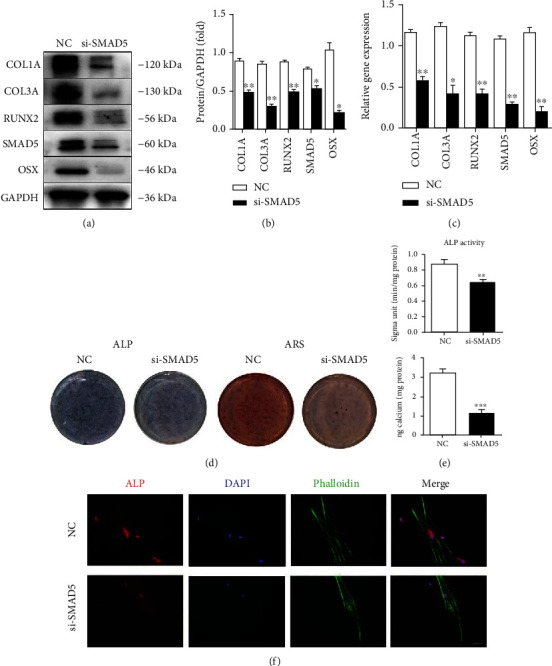
SMAD5 regulates the osteoblastic differentiation ability of PDLSCs. (a)–(c) Western blot and RT-qPCR showed that osteogenic markers (RUNX2, OSX, COL1A, and COL3A) were downregulated in the SMAD5 siRNA-treated PDLSCs (si-SMAD5 group). (d, e) Silence of SMAD5 reduced the ALP activity of PDLSCs 7 days after osteogenic induction. ARS showed the mineralization of PDLSCs was decreased significantly in the si-SMAD5 group after osteogenic induction for 14 days. (f) Immunofluorescence experiments confirmed the decreased expression of ALP in the si-SMAD5 group. ^∗^*P* < 0.05, ^∗∗^*P* < 0.01, and ^∗∗∗^*P* < 0.001.

PDLSCs were transfected with NC or SMAD5 siRNA (si-SMAD5). Western blot and RT-PCR results showed SMAD5 and biomarkers of osteogenesis (RUNX2, OSX, COL1A, and COL3A) were downregulated (Figures 4(a)–4(c)).

The osteogenic induction medium was used for culturing two groups of cells, respectively. At 14 days, the CPC assay and ARS results showed that the mineralization nodules and calcium content in the si-SMAD5 group were significantly reduced (*P* < 0.001). After seven days, to early observe the osteogenic differentiation ability of PDLSCs, ALP activity was performed. The results proved that the expression of ALP in the si-SMAD5 group was reduced, and the ALP activity decreased significantly (*P* < 0.001) (Figures 4(d) and 4(e)). Immunofluorescence experiments confirmed that the expression of ALP in PDLSCs in the si-SMAD5 group was significantly downregulated (Figure 4(f)).

### 3.5. Overexpression of miR-4781-3p Decreases Osteoblastic Differentiation Tendency of PDLSCs

**Figure 5:**
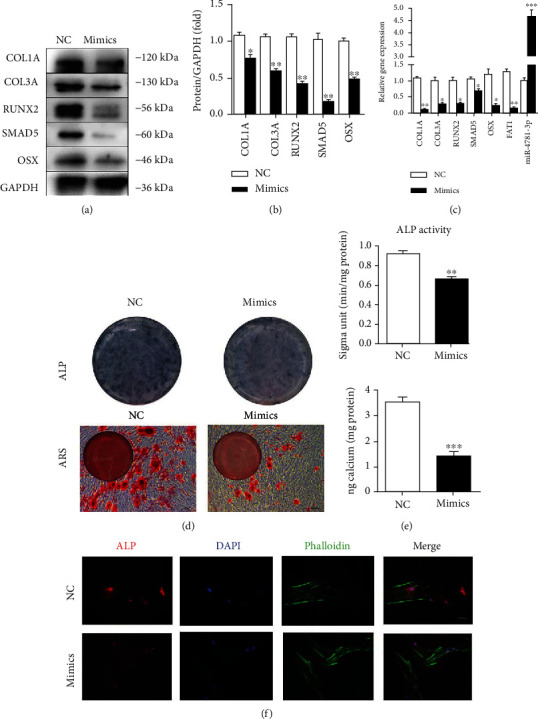
Overexpression of miR-4781-3p decreases osteoblastic differentiation tendency of PDLSCs. (a)–(c) The results of Western blot and RT-qPCR showed SMAD5 and osteogenic markers (RUNX2, OSX, COL1A, and COL3A) were downregulated in miR-4781-3p mimic transfected PDLSCs (mimics group). In addition, RT-qPCR results showed that the expression of *circFAT1* was downregulated, and *miR-4781-3p* was upregulated in the mimics group. (d, e) Overexpression of miR-4781-3p reduced the ALP activity of PDLSCs 7 days after osteogenic induction. ARS showed the mineralization of PDLSCs was decreased significantly in the mimic group after osteogenic induction for 14 days. (f) Immunofluorescence experiments confirmed the decreased expression of ALP in the mimics group. ^∗^*P* < 0.05, ^∗∗^*P* < 0.01, and ^∗∗∗^*P* < 0.001.

PDLSCs were transfected with NC for mimics or miR-4781-3p mimics (mimics), respectively. Western blot and RT-qPCR showed that SMAD5 and biomarkers of osteogenesis (RUNX2, OSX, COL1A, and COL3A) were reduced. In addition, RT-PCR results displayed less expression of *circFAT1* and increased expression of *miR-4781-3p* (Figures 5(a)–5(c)).

The mineralization induction medium was used for culturing two groups of PDLSCs, and ARS and CPC assay was performed at 14 days. It was found that the mineralization nodules and calcium content of the mimics group were significantly reduced (*P* < 0.001). After a week, the ALP activity was detected, less expression of ALP was found in the mimics group, and the ALP activity was downregulated significantly (*P* < 0.001) (Figures 5(d) and 5(e)). Immunofluorescence experiments confirmed that the expression of ALP in PDLSCs in the mimics group was significantly reduced (Figure 5(f)).

### 3.6. Knockdown of miR-4781-3p Increases Osteoblastic Differentiation Tendency of PDLSCs

**Figure 6:**
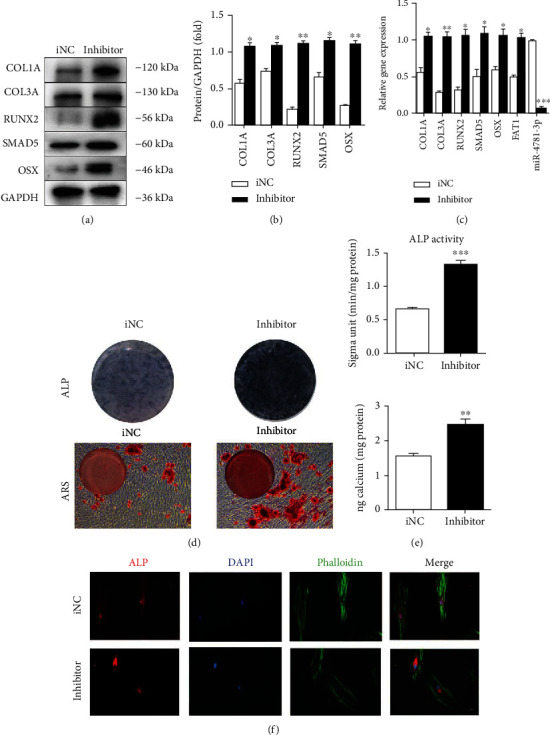
Knockdown of miR-4781-3p increases osteoblastic differentiation tendency of PDLSCs. (a)–(c) The results of Western blot and RT-qPCR showed SMAD5 and osteogenic markers (RUNX2, OSX, COL1A, and COL3A) were upregulated in the miR-4781-3p inhibitor transfected PDLSCs (inhibitor group). In addition, RT-qPCR results showed that the expression of *circFAT1* was upregulated, and *miR-4781-3p* was downregulated in the inhibitor group. (d, e) Knockdown of miR-4781-3p increased the ALP activity of PDLSCs 7 days after osteogenic induction. ARS showed the mineralization of PDLSCs was added significantly in the inhibitor group after osteogenic induction for 14 days. (f) Immunofluorescence experiments confirmed the increased expression of ALP in the inhibitor group. ^∗^*P* < 0.05, ^∗∗^*P* < 0.01, and ^∗∗∗^*P* < 0.001.

PDLSCs were transfected with negative inhibitor control (iNC) or miR-4781-3p inhibitor (inhibitor), respectively. Western blot and RT-qPCR showed that the expression of SMAD5 and biomarkers of osteogenesis (RUNX2, OSX, COL1A, and COL3A) increased. In addition, RT-qPCR results showed that the *circFAT1* expression was increased while the expression of *miR-4781-3p* was decreased (Figures 6(a)–6(c)).

The mineralization induction medium was used for culturing two groups of PDLSCs. ARS and CPC determination were performed on the 14th day. The results showed that mineralized nodules and calcium content increased significantly in the inhibitor group (*P* < 0.001). ALP activity was detected after a week, and the results showed that an increasing ALP expression in the inhibitor group and ALP activity was also upregulated (*P* < 0.001) (Figures 6(d) and 6(e)). Immunofluorescence analysis confirmed that the ALP expression was promoted in the inhibitor group (Figure 6(f)).

### 3.7. circFAT1 Acting as a miRNA Sponge for miR-4781-3p by Targeting SMAD5

**Figure 7:**
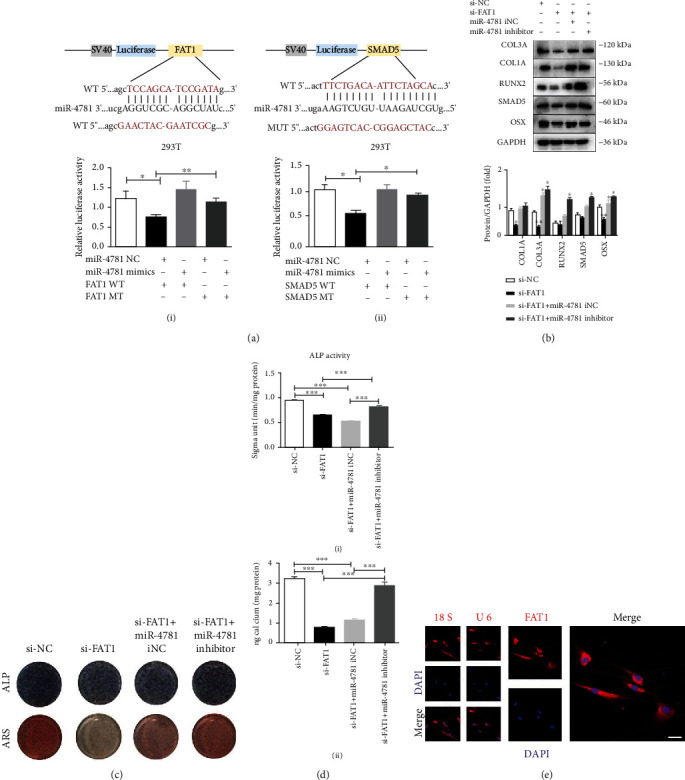
circFAT1 acting as a miRNA sponge for miR-4781-3p by targeting SMAD5. (a) I: Dual-luciferase reporter gene assay was used to verify the binding sites between circFAT1 and miR-4781-3p. II: Dual-luciferase reporter gene assay was used to verify the binding sites between SMAD5 and miR-4781-3p. (b) Rescue experiment results showed that miR-4781 inhibitor could reverse the inhibition of circFAT1 siRNA on the osteogenic ability of PDLSCs. The expression level of SMAD5 was also reversed by miR-4781 inhibitor. (c) After seven days, less expression of ALP was found in the si-FAT1 group and si-FAT1 + miR-4781 iNC group, while the expression of ALP increased in the si-FAT1 + inhibitor group. The mineralization nodules in the si-FAT1 group and si-FAT1 + miR-4781 iNC group decreased. (d) I: ALP activity showed the same trend. II: Mineralized nodules were significantly increased in the si-FAT1 + inhibitor group, and calcium content was significantly increased (*P* < 0.001). (e) FISH experiments proved that circFAT1 were mostly located in the cytoplasm, 18S and U6 were the internal control. (Scale bar: 25 *μ*m). ^∗^*P* < 0.05, ^∗∗^*P* < 0.01, and ^∗∗∗^*P* < 0.001.

To verify the sponge effect of circFAT1 as miR-4781-3p competitively combining SMAD5, circFAT1 wild-type (FAT1 WT) and mutant plasmid (FAT1 MT) were constructed, respectively. By cotransfection with miR-4781 negative control (NC) and miR-4781 mimics (mimics), dual-luciferase reporter assay showed that miR-4781 mimics significantly inhibited the luciferase activity of circFAT1 wild-type reporter gene and confirm the existence of binding sites (Figure 7(a)). SMAD5 wild-type (SMAD5 WT) and mutant plasmid (SMAD5 MT) were constructed, respectively. By cotransfection with miR-4781 negative control (NC) and miR-4781 mimics (mimics), dual-luciferase reporter assay showed that miR-4781 mimics significantly inhibited the luciferase activity of SMAD5 wild-type reporter gene and confirm the existence of binding sites between SMAD5 and miR-4781 (Figure 7(a)).

Meanwhile, a rescue experiment was carried out to further verify. The western blot results revealed that inhibiting miR-4781 could reverse the inhibitory effect of circFAT1 siRNA on the osteogenic ability of PDLSCs. And the miR-4781 inhibitor reversed the expression of SMAD5 (Figure 7(b)).

The osteogenic induction medium was used for culturing four groups of cells, respectively. CPC assay and ARS were performed at 14 days, and it was found that the mineralization nodules in the si-FAT1 group and si-FAT1 + miR-4781 iNC group decreased. Mineralized nodules were significantly increased in the si-FAT1 + inhibitor group, and calcium content was significantly increased (*P* < 0.001). After seven days, less expression of ALP was found in the si-FAT1 group and si-FAT1 + miR-4781 iNC group, while the expression of ALP increased in the si-FAT1 + inhibitor group. ALP activity showed the same trend (Figures 7(c) and 7(d)). FISH experiments proved that circFAT1 was mostly located in the cytoplasm, and 18S and U6 were detected as the internal control (Figure 7(e)).

### 3.8. Silence of circFAT1 Inhibits the Osteogenic Differentiation Ability of PDLSCs *In Vivo*

**Figure 8:**
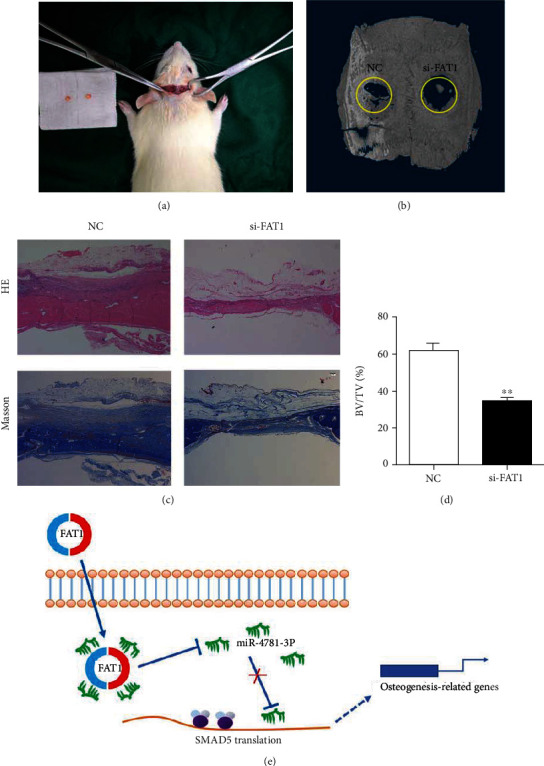
Silence of circFAT1 inhibits the osteogenic differentiation ability of PDLSCs in vivo. (a) The skull defect model of SD rats was constructed. (b) 3D reconstruction image of the rat skull showed that bone regeneration in si-FAT1 group was inhibited. (c) H&E staining and Masson staining results both indicated that the new bone mass in the si-FAT1 group was significantly reduced. (Scale bar: 50 *μ*m) (d) The bone volume fraction (BV/TV) was decreased in the si-FAT1 group. (e) Experimental mechanism diagram: circFAT1 may regulate PDLSC bone regeneration by targeting SMAD5 through acting as a sponge for miR-4781-3p. ^∗^*P* < 0.05, ^∗∗^*P* < 0.01, and ^∗∗∗^*P* < 0.001.

To further understand the role of circFAT1, the skull defect model of SD rats was constructed (Figure 8(a)). PDLSC cell mass transfected with NC or circFAT1 siRNA (si-FAT1) was placed, respectively, and the tissue samples were taken eight weeks later. 3D reconstruction image of the rat skull showed that bone regeneration in the si-FAT1 group was inhibited (Figure 8(b)). H&E and Masson staining results indicated that the new bone mass in the si-FAT1 group was significantly reduced. The bone volume fraction was decreased in the si-FAT1 group by calculating the ratio of bone volume and tissue volume (BV/TV) (*P* < 0.01) (Figures 8(c) and 8(d)). Therefore, as shown in the pattern diagram, circFAT1 may regulate PDLSC osteoblastic regeneration by targeting SMAD5 through acting as a sponge for miR-4781-3p (Figure 8(e)).

## 4. Discussion

As one of the most common oral inflammatory diseases, periodontitis is often related to human tooth loss. For periodontal tissue with complex structure, tissue regeneration and stable microenvironment are challenging for currently available treatments. In regenerative therapy, since MSCs can be obtained from various tissues, stem cell therapy has attracted more and more attention. PDLSCs are considered the best cell source for periodontal tissue regeneration [33]. In our previous study, LncNEAT1 may target miR-214-5p/SMAD4 to regulate the cementogenic differentiation of PDLSCs (preprint) [34]. Seo et al. [35] found that PDLSCs can create a cementum/periodontal ligament-like structure in vivo. Therefore, PDLSCs may be the key to periodontal tissue regeneration [35]. Although odontogenic stem cells can differentiate into various compartments, the number of cells that can perform the required functions is limited. Therefore, it is vital to induce PDLSCs to differentiate into osteoblasts and cementoblasts [36].

SMAD5 is considered to be involved in regulating osteogenic differentiation [37]. After bone morphogenetic protein 2 (BMP-2) binds to the receptor in the bone morphogenetic protein (BMP) pathway, SMAD5 is activated by phosphorylation and binds to SMAD1 and SMAD8 to form polymers and then transferred to the nucleus and positively regulated the transcription of osteogenic genes [38]. This regulatory effect is mainly achieved by interacting with various transcription regulators, targeting several *cis*-acting promoter elements among osteogenic genes like ALP and osteocalcin (OCN). It is worth noting that the upregulation of RUNX2, as a SMAD5 transcription factor, largely determines this effect [39]. Many studies have confirmed that miRNAs targeting SMAD5 inhibit osteogenic differentiation. In this study, we demonstrated the direct regulatory effect of miR-4781-3p on SMAD5. After SMAD5 knockout, the osteogenic differentiation potential of PDLSCs was limited, and the overexpression of miR-4781-3p could reverse this effect.

Recent studies have shown that circRNAs participate in various biological processes such as miRNA sponges, protein binding regulation, and gene transcription and may regulate multiple diseases, including periodontitis. Both *in vitro* [29, 40, 41] and *in vivo* [42, 43] studies have confirmed that circRNAs are involved in regulating the osteogenic differentiation of PDLSCs. In the process of PDLSCs' osteoblastic induction and mechanical stimulation, specific circRNAs were detected upregulated or downregulated. circCDK8 was proved to impair the osteogenesis of PDLSCs under hypoxia [29]. It is found that circRNA function through the network relationship between circRNAs and miRNAs. CircRNA 3140 targets miR-21, and circRNA 436 may act as a sponge for miR-107 and miR-335 [41, 44]. CircMAP3K11 may promote the proliferation of PDLSCs and inhibit their apoptosis by acting as a sponge for miR-511-3p [42]. CircCDR1as acts as a sponge for miR-7 in PDLSC proliferation and differentiation, affecting ERK or MAPK signaling pathways [43]. In addition, circRNAs may also regulate bone formation by regulating extracellular matrix tissue and cell differentiation and affect the BMP signaling pathway, according to sequencing analysis of osteogenic induced PDLSCs. Gu et al. [44] found that 766 circRNAs were upregulated, and 690 were downregulated in PDLSCs 7 days after osteogenic induction. It is reasonable to believe that the competing endogenous RNA (ceRNA) network seems to be the main mechanism for circRNAs to perform functions during the osteogenesis of PDLSCs [44]. However, it is still necessary to better understand all the potential mechanisms that may regulate circRNAs. Some studies have found circFAT1 can act as a sponge for miR-30a-5p in competitive combination with REEP3, thus influencing the progression of HCC [45], but there is no relevant research on the biological characteristics of circFAT1 and its role in oral diseases. It is worth noting that there seems to be no common circRNAs among different oral cell types. For instance, circ0081572 was found in gingival tissue (not cells) and PDLSCs [39]. These results suggest that circRNAs may be cell-specific in periodontal tissue. Because some circRNAs can change cell behaviors, it is promising to study it as a therapeutic target to regulate stem cell differentiation and optimize periodontal regeneration. Further research on circRNAs is needed to understand their role in periodontal diagnosis and regeneration.

## 5. Conclusions

In this study, SMAD5 was found positively regulated the osteogenic differentiation of PDLSCs. miR-4781-3p may participate in inhibiting the protein translation of SMAD5, blocking its expression, and then reducing osteogenic differentiation of PDLSCs. The binding sites between SMAD5 and miR-4781-3p and between circFAT1 and miR-4781-3p were confirmed by analysis of dual-luciferase reporter assay, respectively. The functional relationship between the three was verified by rescue experiments. *In vivo*, inhibiting the expression of circFAT1 reduced the bone regeneration of rat skull defect. Therefore, combined with *in vivo* and *in vitro* studies, we could conclude that circFAT1 may be involved in the regulation of PDLSCs' osteogenic differentiation through the ceRNA network of miR-4781-3p/SMAD5. Whether there are other regulatory mechanisms remains to be further explored. Our research may be helpful to explore the role of circFAT1 in periodontal regeneration and verify the mechanism of its possible regulation of SMAD5 through sponging with miR-4781-3p, aiming to investigate the potential target of PDLSCs mediated periodontal bone regeneration.

## Figures and Tables

**Table 1:** Sense and antisense primers for RT-qPCR.

Genes	Primers	Sequences (5′-3′)
*GAPDH*	Forward	TCACCAGGGCTGCCATCTGCTCTC
	Reverse	TTGCAGTGGCAAAGTGGAGATTGTTG
*COL1A*	Forward	TCTGACTGGAAGAGCGGAGAG
	Reverse	GAGTGGGGAACACACAGGTCT
*COL3A*	Forward	CTGTGAATCATGCCCTACTGGTC
	Reverse	AAGCCTCTGTGTCCTTTCATACC
*RUNX2*	Forward	TCTTAGAACAAATTCTGCCCTTT
	Reverse	TGCTTTGGTCTTGAAATCACA
*OSX*	Forward	GCCTACTTACCCGTCTGACTTT
	Reverse	GCCCACTATTGCCAACTGC
*SMAD5*	Forward	ACCGCACATGCCACAAAAC
	Reverse	CAGGGGAAGGAGGATAGGG
*FAT1*	Forward	GATGAGGACGCCAGAAGAGA
	Reverse	CAAATGTCTCCCCATTGCTT
*miR-4781-3p*	Forward	CGCGCGGGGAACCCGC
	Reverse	AGTGCAGGGTCCGAGGTATT

## Data Availability

The raw data used to support the findings of this study are available from the corresponding author upon request.
